# Partner fidelity, not geography, drives co-diversification of gut microbiota with hominids

**DOI:** 10.1098/rsbl.2024.0454

**Published:** 2025-01-29

**Authors:** Andrew H. Moeller

**Affiliations:** ^1^ Department of Ecology and Evolutionary Biology, Princeton University, Princeton, NJ 08544, USA

**Keywords:** microbiome, metagenome, symbiosis, isolation by distance, host specificity, chimpanzees

## Abstract

Bacterial strains that inhabit the gastrointestinal tracts of hominids have diversified in parallel (co-diversified) with their host species. The extent to which co-diversification has been mediated by partner fidelity between strains and hosts or by geographical distance between hosts is not clear due to a lack of strain-level data from clades of hosts with unconfounded phylogenetic relationships and geographical distributions. Here, I tested these competing hypotheses through meta-analyses of 7121 gut bacterial genomes assembled from wild-living ape species and subspecies sampled throughout their ranges in equatorial Africa. Across the gut bacterial phylogeny, strain diversification was more strongly associated with host phylogeny than with geography. In total, approximately 14% of the branch length of the gut bacterial phylogeny showed significant evidence of co-diversification independent of geography, whereas only approximately 4% showed significant evidence of diversification associated with geography independent of host phylogeny. Geographically co-occurring heterospecific hosts (*Pan* and *Gorilla*) universally maintained distinct co-diversified bacterial strains. Strains whose diversification was associated with geography independent of host phylogeny included clades of Proteobacteria known to adopt free-living lifestyles (e.g. *Escherichia*). These results show that co-diversification of gut bacterial strains with hominids has been driven primarily by fidelity of strains to host lineages rather than geography.

## 1. Background

In primates, the phylogenetic histories of multiple gut bacterial strains parallel those of their host species [1–5], consistent with evolutionarily ancient host species-specific symbioses, but the processes that drive this co-diversification are poorly understood. Co-diversification may result from partner fidelity mediated by fitness advantages for endogenous gut microbiota (GM) strains conferred by host traits, which could include immune, dietary or physiological traits as well as social behaviours that transmit strains within (but not between) host populations [6–9]. Alternatively, co-diversification could be driven by limits on gene flow between GM strains of different host species imposed by geographical distance, which could operate in the absence of selective processes within hosts or host populations.

These alternative hypotheses have not been tested because the geographical distributions and phylogenetic histories of well-sampled hosts analysed to date have been confounded. For example, the genealogical relationships of extant human populations are confounded with the populations’ geographical distributions, such that the extent to which co-diversification between GM strains and human populations has been driven by partner fidelity or geography has remained controversial [2,10,11]. Similarly, recent studies of co-diversification between gut microbiota and hominid species have focused only on subsets of these hosts’ geographical ranges [3–5], precluding high-powered tests for independent effects of host phylogenetic divergence and geographical distance on the diversification of GM strains.

African apes provide a natural experiment with which to resolve the independent effects of host phylogeny and geography on the diversification of GM strains because the hosts’ geographical distributions are in some cases independent of phylogenetic relationships. Previous work used broad geographical sampling of chimpanzees, bonobos and gorillas—including sympatric and allopatric populations—to quantify independent effects of host phylogeny and geographical distance on the taxonomic composition of the gut microbiota (as assessed by 16S rDNA amplicon sequencing; [12]). However, this broad sampling design has not been used to assess the drivers of GM-strain diversification due to incomplete strain-level GM datasets from these hosts. Recent work has generated metagenomic data and repositories of metagenome-assembled genomes (MAGs) from diverse populations of non-human African apes spanning the extant geographical ranges of these hosts [4,5,13–15]. Here, I conducted meta-analyses of African-ape-derived MAGs to quantify the independent contributions of host phylogenetic divergence and geographical distance to the diversification of hominid GM strains. Analyses showed that partner fidelity has maintained co-diversifying GM strains within hominid lineages irrespective of geography.

## 2. Material and methods

### (a) Taxonomic classification and phylogenetic analyses of African-ape gut bacterial genomes

MAGs from the gut microbiotas of three subspecies of chimpanzees (*Pan troglodytes verus, Pan troglodytes schweinfurthii* and *P. t. troglodytes*) sampled at 10 sites, bonobos (*Pan paniscus*) sampled at seven sites, western and eastern gorillas (*Gorilla gorilla* and *Gorilla beringei*, respectively) sampled at two sites were retrieved from MAG databases compiled by previous studies [4,5], which generated and curated MAGs from de novo sequencing and available datasets [13–15]. Due to humans’ complex migration histories (e.g. international travel) relative to those of other African apes, these analyses focused on non-human African apes. All MAGs were classified to taxonomic ranks using the genome taxonomy database toolkit v. 2.3.2 using the commands identify, align and classify with default settings [16]. MAGs estimated as greater than 50% complete and less than 5% contaminated by CheckM were used for downstream analyses [17]. The alignments of genes from each MAG to the reference set of bac120 single-copy core genes were used for phylogenetic analyses in IQTree2 v. 2.2.2.6 with the following parameters: --seed 0 -ntmax 24 -B 1000 -bnni -alrt 1000 -mset WAG,LG [18].

### (b) Host geographical and phylogenetic distances

A phylogenetic tree of the African ape species and subspecies was obtained from previous work [19]. Phylogenetic distances between species and subspecies were calculated using tip_tip_distances in Python’s scikit-bio v. 0.5.9. The geographical distances among sampling sites were calculated using the haversine formula and the global positioning system coordinates of each sampling location.

### (c) Tests for strain diversification associated with host phylogenetic or geographical distances

Tests for diversification of clades in the bacterial phylogeny associated with either host phylogenetic or geographical distances were conducted using a previously developed approach [4]. In this workflow, available at https://github.com/CUMoellerLab/codiv-tools, each node in the distal tenth of the bacterial phylogeny was tested for diversification associated with either host phylogenetic or geographical distances using an extension of a test for co-speciation based on Mantel permutation tests [20]. Here, distal tenth refers to clades for which the maximum tip-to-tip distances within the clade was 10% or less of the phylogeny’s maximum tip-to-tip distance. Tests were restricted to the distal tenth of the bacterial phylogeny based on previously published molecular clocks supporting that this window corresponds to the timescales of African-ape diversification [4]. For each node, this analysis calculated a Mantel’s correlation coefficient between the bacterial tip-to-tip distances and either the corresponding host tip-to-tip distances (for tests for association with host phylogeny) or the geographical distances between locations at which the hosts were sampled (for tests for association with geography). For each node tested, the observed Mantel’s correlation coefficients were compared with null distributions obtained by 1000 random permutations of the mapping between the symbiont phylogenetic distance matrix and the reference distance matrix, the latter of which contained either host phylogenetic or geographical distances. These analyses yielded non-parametric *p*-values for each node indicating the significance of association between bacterial diversification within the clade and either host phylogenetic distances or geographical distances. To account for multiple testing, *p*-values were corrected with the Benjamini–Hochberg method. For each symbiont node tested, the difference between Mantel’s *r* with host phylogenetic distances and Mantel’s *r* with host geographical distances was used to assess relative associations with the hosts’ phylogenetic distances or geographical distances, with positive and negative values indicating a greater degree of association with host phylogenetic or geographical distances, respectively.

### (d) Classification of bacterial lifestyles

Traits of bacterial taxa were compiled from *Bergey’s manual of systematics of Archaea and Bacteria* [21] and previous work [22]. Trait classifications used for tests for association with the degree of GM-strain partner fidelity are presented in electronic supplementary material, table S3.

### (e) Statistical analyses and visualizations

Centroids of taxa with respect to Mantel’s *r* coefficients were calculated in Python v. 3.11.8. Phylogenetic trees were visualized using empress v. 0.1 [23]. Scatter plots, box plots and histograms were generated with ‘ggplot2’ v. 3.4.4 [24]. Regression analyses were conducted in R v. 4.3.2 using the lm function. Phylogenetic trees were filtered using the drop.tip function and phylogenetic analysis of variance (ANOVA) was performed using the phylANOVA function in the R package ‘phytools’ 2.1-1 [25]. Sequence reads from metagenomes were filtered for quality with cutadapt [26] v. 4.9 then mapped against the MAG set dereplicated at 95% average nucleotide identity using minimap2 [27] (v. 2.28) with the setting -ax map-iclr.

## 3. Results

### (a) Sampling from African apes with independent geographical distributions and phylogenetic relationships

**Figure 1.**
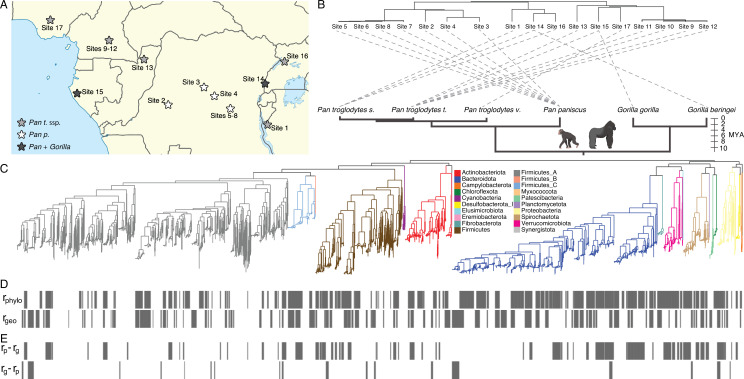
Co-diversification of GM strains with host phylogeny or geography. (*a*) Map shows locations of sampling sites throughout equatorial Africa. (*b*) Tanglegram shows a lack of correspondence between neighbour-joining tree of geographical distances among sites (top tree) and the host phylogeny (bottom tree). Dashed lines connect hosts to the sites at which they were sampled. (*c*) Phylogeny shows relationships among gut bacterial strains recovered from the host species shown in (*b*). Branches are coloured by phylum as indicated by the inset. (*d*) Vertical bars mark clades in (*c*) whose within clade phylogenetic distances show significant associations (*r* > 0.7, *p*‐value < 0.05) with host phylogenetic distances (*r*_phylo_; top row) or geographical distances (*r*_geo_; bottom row). (*e*) Vertical bars mark clades in (*c*) whose within clade phylogenetic distances show significant associations with host phylogenetic distances independently of geography distances (*r*_phylo_−*r*_geo_ > 0.5; *p*‐value < 0.05 for *r*_phylo_ but >0.05 for *r*_geo_; top row) or geographical distances independently of host phylogenetic distances (*r*_geo_−*r*_phylo_ > 0.5; *p*‐value < 0.05 for *r*_geo_ but >0.05 for *r*_phylo_; bottom row).

To quantify the contributions of partner fidelity (host phylogeny) and geography to the diversification of hominids’ gut bacterial strains, I analysed African ape-derived MAGs from 134 hosts belonging to six host species/subspecies and sampled at 17 sites (11–55 individual hosts per subspecies; figure 1*a*). The number of hosts sampled per species and site are presented in electronic supplementary material, tables S1 and S2. Phylogenetic and geographical distances were largely independent in this clade of hosts (figure 1*b*), enabling independent quantification of the relative contributions of partner fidelity and geography to the diversification of the hosts’ GM strains.

### (b) A strain-resolved phylogeny of African-ape gut microbiota

**Figure 2.**
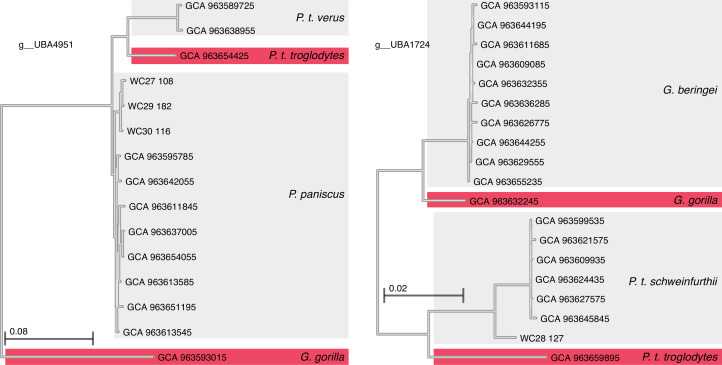
Examples of host-species specificity of co-diversified GM strains in sympatric chimpanzees and gorillas. Phylogenies show relationships of GM strains (unclassified genera UBA4951 and UBA1724) from *Pan* and *Gorilla* hosts sampled in sympatry and allopatry. GM strains sampled from sympatric populations of *Pan* and *Gorilla* are highlighted with red backgrounds. Of the 17 co-diversifying clades that contained metagenome-assembled genomes (MAGs) from *Pan* and *Gorilla* sampled in sympatry, all 17 showed the pattern in which MAGs from sympatric *Pan* and *Gorilla* were more closely related to MAGs from conspecific hosts sampled at different field sites than to MAGs from heterospecific hosts sampled in sympatry.

I inferred a phylogeny based on single-copy core genes from 7121 MAGs from chimpanzees (*P.t.s*.: 2026 MAGs; *P.t.t*: 728 MAGs; *P.t.v*.: 2181 MAGs), bonobos (*P.p*.: 1459 MAGs) and gorillas (*G.b.*: 591 MAGs; *G.g.*: 136 MAGs) (electronic supplementary material, table S2). The number of MAGs per host species is presented in electronic supplementary material, table S1. For each host species, a median of greater than 54% of reads per sample mapped to the reference MAG set, ranging from 54.36% to 84.60% among host species (electronic supplementary material, table S1), indicating that the MAGs captured most of the metagenomic sequences from the faecal samples. Phylogenetic analyses based on single-copy core genes of these MAGs recovered the monophyly of bacterial phyla known to inhabit hominid gut microbiota (figure 2*a*).

### (c) Co-diversification with host phylogeny independent of geography

Previous work has shown that multiple gut bacterial lineages have co-diversified with primate species [4,5], but due to the limited geographical sampling in these studies, it was not possible to test whether co-diversification was mediated by partner fidelity of bacterial lineages to hosts (host phylogeny) or by geographical separation between hosts. Here, permutation tests generated Mantel’s *r* correlation coefficients and non-parametric *p*-values indicating the association between bacterial phylogenetic distances and host phylogeny or geography for each node in the distal tenth of the bacterial phylogeny. In total, 229 and 140 clades showed evidence of diversification paralleling host phylogeny or geography, respectively, based on the thresholds of *r* > 0.7 and Benjamini–Hochberg corrected *p*‐value < 0.05 (following thresholds employed by previous work; figure 1*d*; electronic supplementary material, table S3).

For each node, the difference between Mantel’s *r* correlation coefficients derived from tests based on host phylogeny (*r*_phylo_) and geography (*r*_geo_) provided a statistic (*r*_phylo_*−r*_geo_) with which to identify clades of strains whose phylogenetic relationships were associated with host phylogenetic distances independent of geographical distances or with geographical distances independent of host phylogenetic distances. Significant nodes for which *r*_phylo_−*r*_geo_ was > 0.5 or < −0.5 were classified as associated with host phylogeny independent of geography or geography independent of host phylogeny, respectively. This threshold was chosen to identify clades showing the most extreme differences between Mantel’s correlations with host phylogeny and geography. These analyses identified 87 clades and 23 clades associated with host phylogeny independent of geography or geography independent of host phylogeny, respectively (figure 1*e*). In total, clades associated with host phylogeny independent of geography constituted approximately 13.5% of the branch length in the gut bacterial phylogeny, whereas clades associated with geography independent of host phylogeny constituted only approximately 4.1%. The stronger effects of host phylogeny compared to geography were not sensitive to the specific choice of significance thresholds: distributions of *r*_phylo_ and *r*_geo_ for GM-strain clades confirmed that a greater number of clades showed stronger associations with host phylogeny than with host geography across a range of thresholds (e.g. *r* > 0.7 or *r* > 0.9; electronic supplementary material, figure S1). These results indicate that co-diversification of GM strains with hominids is better explained by host phylogenetic divergence than by geography.

### (d) Maintenance of co-diversified, host species-specific strains in sympatric chimpanzees and gorillas

Comparisons of geographically co-occurring (sympatric) populations of *Pan* and *Gorilla* allowed for strong tests for whether co-diversified GM strains are maintained by partner fidelity or geographical separation. In the absence of partner fidelity, host species-specific strains that have co-diversified with allopatric *Pan* and *Gorilla* would be expected to be exchanged by sympatric populations, which are known to share space and food resources [28]. In contrast to this pattern, for all co-diversified clades (*r* > 0.7 and *p*‐value < 0.05) containing GM strains from sympatric *Pan* and *Gorilla* (17 clades containing MAGs from sympatric heterogeneric hosts, 165 MAGs), GM strains from sympatric hosts were more closely related to GM strains from congeneric hosts sampled in other locations (allopatric) than to GM strains sampled from heterogeneric hosts sampled in sympatry (17/17 clades). Conversely, for all non-co-diversified (*r* > 0.7 or *p*‐value < 0.05) clades showing significant associations with geography (*r* > 0.7 and *p*‐value < 0.05) and containing GM strains from sympatric *Pan* and *Gorilla* (five clades containing MAGs from sympatric heterogeneric hosts, 32 MAGs), GM strains from sympatric heterogeneric hosts were more closely related to one another than to GM strains from allopatric congeneric hosts. Examples of the distinctiveness of co-diversified GM strains in sympatric *Pan* and *Gorilla* are shown in figure 2. These results indicate partner fidelity between co-diversified GM strains and host species in sympatry.

### (e) Diversification associated with geography in a minority of gut bacterial taxa

**Figure 3.**
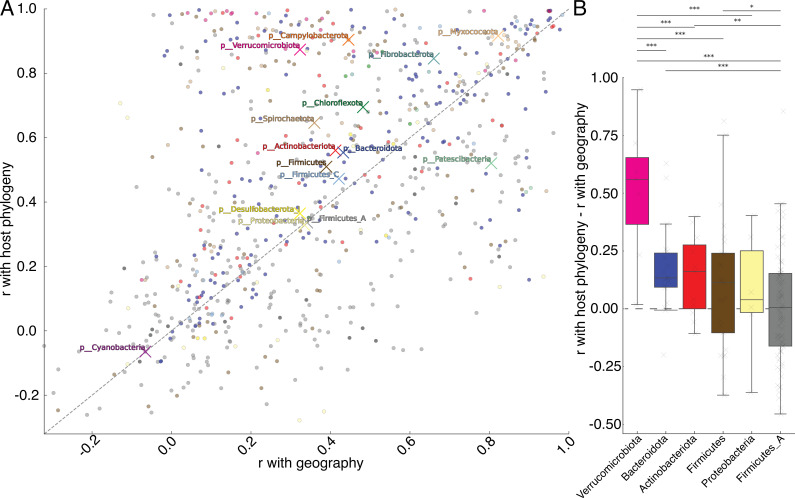
Bacterial taxa differ significantly in the strength of co-diversification with host phylogeny. (*a*) Scatter plot shows the Mantel’s *r* coefficient with geography (*x*-axis) or host phylogeny (*y*-axis) for each bacterial clade (points). Points are coloured by bacterial phylum as in figure 1. Xs mark the branch-length weighted mean values for each bacterial phylum. Dashed line indicates the identity line. (*b*) Box plots show the median and interquartile ranges of the mean strength of association with host phylogeny independent of geography (*r*_phylo_−*r*_geo_) for genera within each bacterial phylum containing five or more genera. Horizontal dashed line marks *r*_phylo_−*r*_geo_ equal to zero. Horizontal solid lines show all significant differences in the per-genus mean values between phyla, based on *t*-tests weighted by unique branch lengths of each genus; Holm-corrected *p*‐value < 0.05*; *p*‐value < 0.01**; *p*‐value < 0.001.

Next, I tested the extent to which clades’ strength of associations with host phylogeny or geography differed significantly among GM taxa. For each bacterial phylum, I calculated the branch-length weighted mean value of Mantel’s *r* correlation coefficients with host phylogenetic distances and geographical distances for all nodes within the phylum. Results showed that the centroids for 12/15 bacterial phyla displayed a higher Mantel’s *r* with host phylogeny than with geography (sign-test *p*‐value = 0.0176; figure 3*a*). Similarly, assessing these values for each genus independently revealed that strains within 99 out of 156 genera showed stronger associations with host phylogeny than with geography (electronic supplementary material, figure S2; sign-test *p*‐value = 0.001). Bacterial phyla differed significantly in the branch-length weighted average values for genera within the phyla. Strains within genera of Verrucomicrobiota, Bacteroidota and Actinobacteroidota consistently showed stronger associations with host phylogeny independent of geography than did strains within genera of Firmicutes_A, Firmicutes and Proteobacteria (figure 3*b*; Holm-corrected weighted *t*‐test *p*-values < 0.01, electronic supplementary material, table S3).

Previous work has suggested that the degree to which GM taxa in mammals display specificity to host lineages varies as a function of transmission mode and bacterial lifestyle [22,29]. For instance, aerobic and spore-forming lifestyles have been associated with horizontal transmission and generalism [22,29]. Here, the maximum *r*_phylo_*−r*_geo_ for strains did not differ significantly between aerobes and anaerobes or between spore formers and non-spore formers after controlling for bacterial phylogenetic history (phylogenetic ANOVA *p*-values > 0.05). However, the bacterial taxa that displayed the strongest evidence of diversification driven by geography independent of host phylogeny included *Escherichia*, a genus of oxygen-tolerant Proteobacteria that adopt both free-living and host-associated lifestyles. Similarly, *Nanosyncoccus*—a genus containing parasitic lineages with highly reduced genomes (approx. 700 kb) [30]—showed the strongest degree of diversification driven by geography independent of host phylogeny of any bacterial genus, suggesting horizontal transmission of this host-restricted taxon. Cumulatively, these results indicate that the diversification of a subset of GM taxa has been mediated by geographical separation of hosts, but that co-diversification with host lineages has resulted primarily from partner fidelity independent of geographical effects.

## 4. Conclusion

Analyses of gut bacterial genome sequences from a diversity of wild African-ape populations showed that host geographical distributions cannot explain the parallels between GM-strain and hominid phylogenies. Although diversification of a minority of bacterial strains was associated with geography—a finding that was overlooked by previous work that did not assess gut bacterial diversity across the full extent of hominid geographical ranges [4,5]—co-diversification between symbiont and host lineages has been primarily driven by partner fidelity.

A limitation of this study included incomplete sampling of host populations. Mapping analyses indicated that most of the metagenome was represented in our MAG set (median mappability of greater than 54–85%, depending on the host species), and the MAG set included representatives from 20 bacterial phyla. However, some bacterial genomes present in the samples were likely not assembled and therefore not included in the phylogenetic analyses. Similarly, increasing the number of host individuals sampled (which here ranged from 11 to 55 per host subspecies) would likely lead to the inclusion of additional bacterial clades. Thus, the numbers of co-diversifying and geographically structured clades identified here represent lower bound estimates.

Another limitation was the lack of information about how the ranges of hominid populations have changed during hominid diversification. Although migration of individual animals between sampling locales assessed here is unlikely to have occurred given the large spatial distance separating host populations, changes in the degree of geographical distance separating host populations over longer timescales would be expected to enhance or attenuate the strength of association between GM strain diversification and geography detected in the present study. For example, studies of captive populations of chimpanzees, which have recently and artificially been moved into sympatry with humans, have shown that close contact with humans can lead to the replacement of a subset of the co-diversified strains of the chimpanzees with related strains from humans [31,32]. However, in the present study, chimpanzees and gorillas living in sympatry maintained distinct sets of strains from co-diversified clades (e.g. figure 2), suggesting that transfer of these strains between hosts living in close spatial proximity in the wild has been rare even in cases of geographical proximity between the host species. The predominant effects of host phylogeny on GM strain diversification are consistent with the view that transmission of co-diversifying GM strains in the wild occurs primarily through social interactions among conspecifics (e.g. grooming, parent–offspring interactions) [6] rather than through shared environments. Cumulatively, these results indicate that co-diversified gut bacterial strains in hominids are transmitted and maintained within host lineages irrespective of geography.

## Data Availability

All data needed to reproduce the results of this study are made available in previous work [4,5]. Code needed to reproduce the analyses is available at [33]. Description of data can be found in electronic supplementary material, tables S1–S3 and at [33]. Supplementary material is available online [34].
